# Minoxidil Induction of VEGF Is Mediated by Inhibition of HIF-Prolyl Hydroxylase

**DOI:** 10.3390/ijms19010053

**Published:** 2017-12-25

**Authors:** Soohwan Yum, Seongkeun Jeong, Dohoon Kim, Sunyoung Lee, Wooseong Kim, Jin-Wook Yoo, Jung-Ae Kim, Oh Sang Kwon, Dae-Duk Kim, Do Sik Min, Yunjin Jung

**Affiliations:** 1 College of Pharmacy, Pusan National University, Busan 609-735, Korea; roy-yum@hanmail.net (S.Y.); finesiecle@gmail.com (S.J.); gumx1234@gmail.com (D.K.); lsy123@nst.re.kr (S.L.); b04420@nate.com (W.K.); jinwook@pusan.ac.kr (J.-W.Y.); 2 College of Pharmacy, Yeungnam University, Gyeongsan 712-749, Korea; jakim@yu.ac.kr; 3 Department of Dermatology, Seoul National University College of Medicine, Seoul National University Hospital, Seoul 110-744, Korea; oskwon@snu.ac.kr; 4 College of Pharmacy and Research Institute of Pharmaceutical Sciences, Seoul National University, Seoul 151-742, Korea; ddkim@snu.ac.kr; 5 Department of Molecular Biology, College of Natural Science, Pusan National University, Busan 609-735, Korea; minds@pusan.ac.kr

**Keywords:** hypoxia inducible factor, minoxidil, vascular endothelial growth factor, angiogenesis, von Hippel–Lindau protein

## Abstract

The topical application of minoxidil may achieve millimolar concentrations in the skin. We investigated whether millimolar minoxidil could induce vascular endothelial growth factor (VEGF), a possible effector for minoxidil-mediated hair growth, and how it occurred at the molecular level. Cell-based experiments were performed to investigate a molecular mechanism underlying the millimolar minoxidil induction of VEGF. The inhibitory effect of minoxidil on hypoxia-inducible factor (HIF) prolyl hydroxylase-2 (PHD-2) was tested by an in vitro von Hippel–Lindau protein (VHL) binding assay. To examine the angiogenic potential of millimolar minoxidil, a chorioallantoic membrane (CAM) assay was used. In human keratinocytes and dermal papilla cells, millimolar minoxidil increased the secretion of VEGF, which was not attenuated by a specific adenosine receptor antagonist that inhibits the micromolar minoxidil induction of VEGF. Millimolar minoxidil induced hypoxia-inducible factor-1α (HIF-1α), and the induction of VEGF was dependent on HIF-1. Moreover, minoxidil applied to the dorsal area of mice increased HIF-1α and VEGF in the skin. In an in vitro VHL binding assay, minoxidil directly inhibited PHD-2, thus preventing the hydroxylation of cellular HIF-1α and VHL-dependent proteasome degradation and resulting in the stabilization of HIF-1α protein. Minoxidil inhibition of PHD-2 was reversed by ascorbate, a cofactor of PHD-2, and the minoxidil induction of cellular HIF-1α was abrogated by the cofactor. Millimolar minoxidil promoted angiogenesis in the CAM assay, an in vivo angiogenic test, and this was nullified by the specific inhibition of VEGF. Our data demonstrate that PHD may be the molecular target for millimolar minoxidil-mediated VEGF induction via HIF-1.

## 1. Introduction

Minoxidil was developed as a treatment for hypertension four decades ago. A common side-effect of the anti-hypertensive drug was hypertrichosis, which attracted clinical researchers involved in the clinical trial. The puzzling observation initiated the development of a topical formulation of minoxidil for the treatment of androgenetic alopecia [1,2]. In 1988, minoxidil was approved by the U.S. Food and Drug Administration for treating baldness in men, and the 2% and 5% products have been marketed [3].

Despite decades-long pharmacologic research, it is still unknown how minoxidil stimulates hair growth. Recently, improving blood supply to the hair follicles was suggested as a mechanism by which minoxidil increases hair growth [4,5]. Physiologically, prominent changes in skin perfusion are entailed in hair follicle cycling, consisting of the resting phase (telogen), the growth phase (anagen), and the regression phase (catagen) [6]. Angiogenic properties are elicited in the epithelial hair bulbs of anagen follicles while, during human hair follicle regression (catagen), some degeneration of the capillary loops within the dermal papilla occurs [7], likely limiting blood supply to hair matrix cells [8]. Importantly, disorders characterized by hair loss, including androgenetic alopecia, are strongly associated with a reduced vascularization of hair follicles [9].

The hair follicle during the anagen phase mainly utilizes vascular endothelial growth factor (VEGF) to maintain proper vasculature around it [8,10]. Accordingly, the minoxidil-mediated induction of VEGF, which occurs via adenosine receptors, may be an important pharmacologic mechanism for its hair growth effect [4,6,11,12].

Hypoxia-inducible factor-1 (HIF-1), a transcription factor composed of HIF-1α and aryl hydrocarbon receptor nuclear translocator (ARNT, HIF-1β), plays a crucial role in regulating cellular homeostasis under hypoxia where oxygen supply is limited [13]. In order to meet the cellular demand for oxygen, HIF-1 transactivates an array of gene-producing proteins that are able to increase oxygen supply, including VEGF, which promotes angiogenesis [12,14,15,16].

The cellular availability of HIF-1α is responsible for HIF-1 activity, which is largely regulated by HIF-prolyl hydroxylases (PHDs) [17]. PHDs are 2-ketoglutarate-dependent hydroxylases that require molecular oxygen (as a co-substrate) and ascorbate (cofactor) to exert their enzymatic activity [16]. Two conserved proline residues on HIF-1α are hydroxylated by PHDs. The post-translational modification is recognized by the ubiquitin E3 ligase von Hippel–Lindau protein (VHL), leading to ubiquitination and subsequent proteasome degradation [18,19,20,21]. This HIF-1α degradation is prevented under hypoxia, in which HIF-1α hydroxylation is limited due to the inhibition of PHD activity.

The molecular mechanism for minoxidil-mediated VEGF increase has mostly been investigated at low micromolar concentrations (10–30 μM) of minoxidil [4,11]. Topically applied minoxidil (2–5%) affords the drug to a millimolar level at the applied site [22,23,24,25]. A recent paper demonstrated that minoxidil concentration in the skin is about 350 μg/g, which likely corresponds to 1–2 mM, 24 h after an application of 5% minoxidil formulation (Rogaine) on human cadaver skin for a permeation study [26]. However, there is no prior report investigating a minoxidil increase of VEGF at the pharmacologically relevant concentration. In this study, we found that millimolar minoxidil increased the secretion of VEGF in human dermal papilla cells (DPCs) and human keratinocytes (HaCaT cells). Minoxidil induction of VEGF is dependent on HIF-1, which occurs by PHD inhibition leading to the prevention of VHL-dependent HIF-1α degradation. Furthermore, millimolar minoxidil promoted in vivo angiogenesis, in which minoxidil-induced VEGF played a critical role.

## 2. Results

### 2.1. Millimolar Minoxidil Increases the Secretion of VEGF in Human Skin Cells

**Figure 1:**
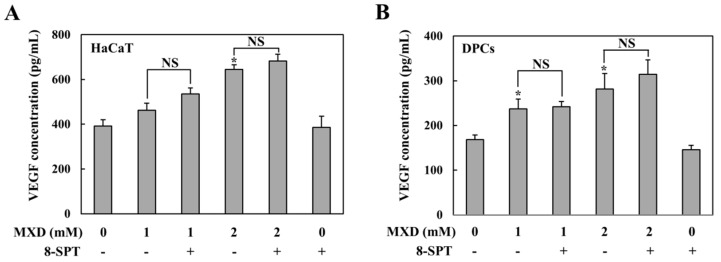
Millimolar minoxidil increases the secretion of vascular endothelial growth factor (VEGF) independently of adenosine receptors. (**A**) Human keratinocytes (HaCaT cells) were treated with minoxidil (MXD) for 10 h in the presence 8-SPT (1 μM), an adenosine receptor antagonist, and VEGF secretion was monitored in cell culture supernatants by ELISA; (**B**) the same experiment was performed in human dermal papilla cells (DPCs). The data represent the mean ± standard error of the mean (SEM) (*n* = 5). * *p* < 0.05 versus untreated group. NS: not significant.

To investigate the pharmacologic mechanism by which minoxidil promotes hair growth in the scalp at a concentration achievable by topical treatment with minoxidil, we examined whether millimolar minoxidil could produce VEGF, a possible key angiogenic factor involved in hair growth [5], in DPCs and HaCaT cells. Cells were treated with minoxidil for 10 h, and the cell culture supernatants were subjected to VEGF ELISA. The same experiment was performed in the presence of 8-SPT, a specific adenosine receptor A1R/A2R antagonist, because low micromolar concentrations of minoxidil induce VEGF via adenosine receptors blockable by 8-SPT [11]. As shown in Figure 1A,B, in both HaCaT cells and DPCs, minoxidil induced VEGF in a concentration-dependent manner and 8-SPT did not affect minoxidil-mediated VEGF induction. The concentration response for 8-SPT in both cell lines is shown in Figure S1.

### 2.2. Millimolar Minoxidil Induction of VEGF Is Dependent on HIF-1

**Figure 2:**
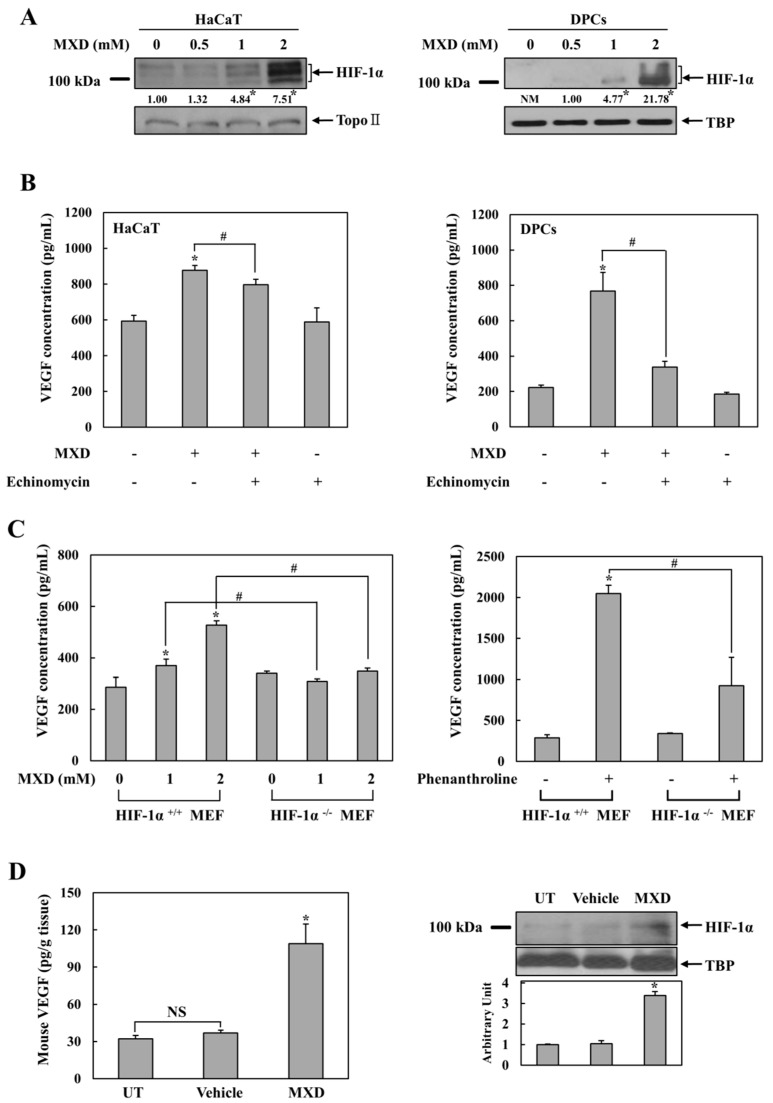
Millimolar minoxidil induction of VEGF is dependent on hypoxia-inducible factor 1 (HIF-1). (**A**) HaCaT cells (Left) or DPCs (Right) were treated with minoxidil (MXD) at various concentrations for 4 h, and HIF-1α in the nuclear extracts was analyzed by Western blot (*n* = 5). * *p* < 0.05 versus untreated group, NM: not measurable, TBP: TATA binding protein, Topo II: Topoisomerase II; (**B**) HaCaT cells (**Left**) or DPCs (**Right**) were treated with MXD (2 mM) for 10 h in the presence of echinomycin (20 nM) and VEGF secretion was monitored in cell culture supernatants by ELISA. * *p* < 0.05 versus untreated group, # *p* < 0.05; (**C**) Wild-type and HIF-1α-deficient mouse embryonic fibroblasts (MEF) were treated with MXD (**Left**) or phenanthroline (200 μM, **Right**) for 10 h and VEGF secretion was monitored in cell culture supernatants. * *p* < 0.05 versus untreated group, # *p* < 0.05; (**D**) MXD (25 μL of 5% Minoxyl solution/cm^2^) or vehicle was applied to the dorsal area of Institute for Cancer Research (ICR) mice for 4 h. Secreted VEGF (**Left**) and nuclear HIF-1α (**Right**) were analyzed in tissue lysates. Western blot results are representative of five independent experiments. * *p* < 0.05 versus untreated or vehicle group, NS: not significant, TBP: TATA binding protein, UT: untreated. The data represent the mean ± SEM (*n* = 5).

The above results suggest that there is a previously unrecognized molecular mechanism for VEGF induction by millimolar minoxidil. Since VEGF is a target gene of the transcription factor HIF-1 [27], we examined whether HIF-1 was involved in VEGF induction by millimolar minoxidil. HaCaT cells and DPCs were treated with 0.5, 1, and 2 mM minoxidil for 4 h, and HIF-1α levels in nuclear extracts were examined. As shown in Figure 2A, a clear induction of HIF-1α protein was observed at 2 mM minoxidil. No HIF-1α induction was observed at minoxidil concentrations less than 0.5 mM. To examine whether minoxidil-mediated VEGF induction was dependent on HIF-1α induction, cells were treated with minoxidil in the presence of echinomycin, a specific HIF-1 inhibitor [28]. As shown in Figure 2B, VEGF induction by minoxidil was significantly reduced by the HIF-1 inhibitor in both cell lines. The concentration response for echinomycin in both cell lines is shown in Figure S1.

To confirm the involvement of HIF-1 in minoxidil-mediated VEGF induction, minoxidil was added to either normal fibroblast cells or matched fibroblast cells with HIF-1α deletion, and VEGF induction was compared. Chemical hypoxia was used as a positive control. As expected, under chemical hypoxia induced by an iron chelator phenanthroline [29], VEGF induction was much greater in normal cells than in HIF-1α-deficient cells (Figure 2C, right panel). In parallel, VEGF induction by minoxidil was substantial in normal cells but negligible in HIF-1α-deficient cells (Figure 2C, left panel), strongly suggesting HIF-1 dependency of minoxidil-mediated VEGF induction. We examined whether the cellular responses to minoxidil were exhibited in vivo upon a topical application of minoxidil at its clinical dose. Commercially available 5% minoxidil solution was applied on the dorsal skin of mice, and VEGF and HIF-1α levels were monitored 4 h after the topical application. The same experiment was performed with the vehicle of the minoxidil solution. As shown in Figure 2D, while the vehicle did not affect the level of VEGF and HIF-1α, minoxidil elevated the level of VEGF (Left panel) and HIF-1α (Right panel) in the mouse skins.

### 2.3. Minoxidil Activation of HIF-1 Occurs by Inhibiting HIF-Prolyl Hdroxylase

**Figure 3:**
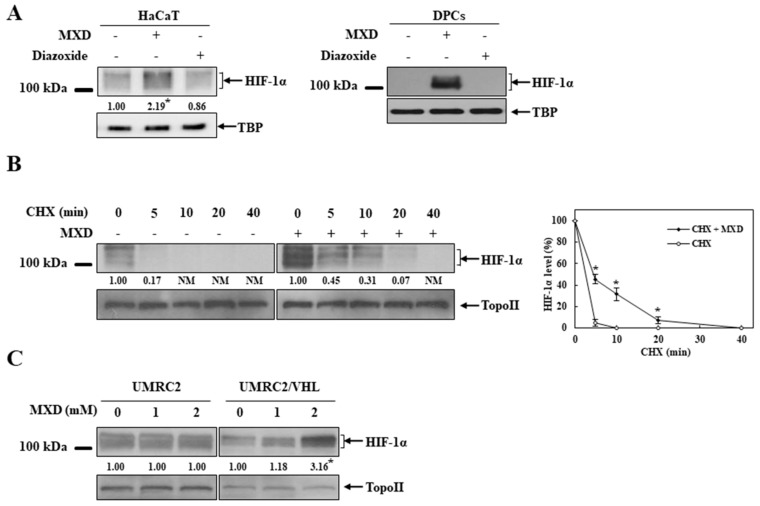
Millimolar minoxidil stabilizes HIF-1α protein. (**A**) HaCaT cells (**Left**) or DPCs (**Right**) were treated with diazoxide (2 mM) or minoxidil (MXD, 2 mM), which are potassium channel activators. HIF-1α in the nuclear extracts was analyzed by Western blot (*n* = 5). * *p* < 0.05 versus untreated group. TBP: TATA binding protein; (**B**) HaCaT cells were either left untreated or were pretreated with MXD (2 mM) for 4 h, followed by an addition of cycloheximide (CHX, 200 µM) for the indicated times. HIF-1α in the nuclear extracts was analyzed by Western blot. The data in the graph represent mean ± SEM (*n* = 5), * *p* < 0.05 versus group treated with CHX alone or initial HIF-1α level. Topo II: Topoisomerase II; (**C**) Renal carcinoma cells that are deficient for von Hippel–Lindau protein (VHL) function (UMRC2) or a clonally selected line with VHL stably expressed (UMRC2/VHL) were treated with MXD for 4 h. HIF-1α in the nuclear extracts was analyzed by Western blot (*n* = 5). * *p* < 0.05 versus untreated group. Western blot results are representative of five independent experiments. Topo II: Topoisomerase II.

**Figure 4:**
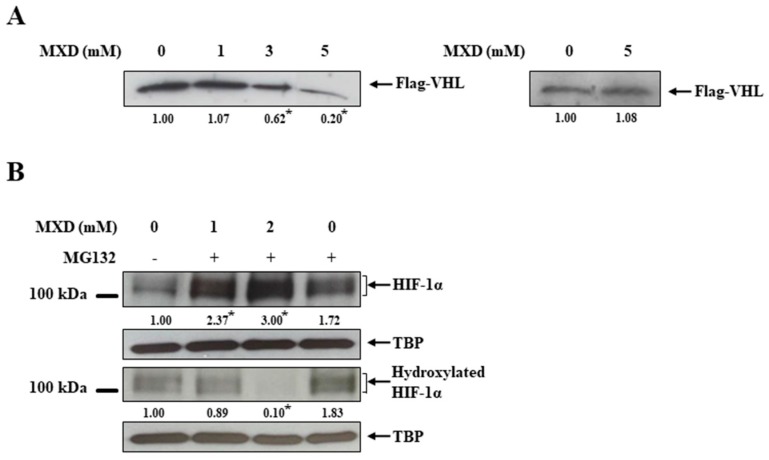
Millimolar minoxidil inhibits HIF prolyl hydroxylase. (**A**) (**Left**) A VHL capture assay was performed in the presence of minoxidil (MXD) at various concentrations, and the resultant blots were probed with the antibody to Flag (VHL). (**Right**) The same assay was repeated utilizing a chemically hydroxylated peptide and MXD (5 mM) (*n* = 5), * *p* < 0.05 versus untreated group; (**B**) HaCaT cells were treated for 4 h with MXD or MG132 (10 μM), a proteasome inhibitor, and were lysed to obtain nuclear extracts. Blots were probed with an anti-HIF-1α antibody and an anti-hydroxylated HIF-1α antibody (*n* = 5). * *p* < 0.05 versus untreated group. Western blot results are representative of five independent experiments. TBP: TATA binding protein.

We next examined the molecular mechanism underlying millimolar minoxidil-mediated HIF-1 activation. To exclude any involvement of potassium channels in the minoxidil induction of HIF-1α, DPCs and HaCaT cells were treated with diazoxide, another potassium channel activator. As shown in Figure 3A, diazoxide did not induce HIF-1α protein. Since HIF-1α is under tight post-translational regulation by protein degradation [19], we examined whether minoxidil affected HIF-1α stability. HaCaT cells pretreated with minoxidil for 4 h were exposed to the protein synthesis inhibitor cycloheximide for the indicated times and the same experiment was performed without minoxidil. The temporal change in the levels of HIF-1α protein was monitored. As shown in Figure 3B, HIF-1α protein disappeared in 5 min in untreated cells while HIF-1α protein still remained in a detectable amount in minoxidil-treated cells 20 min after exposure to cycloheximide, implying that HIF-1α protein was stabilized by minoxidil. HIF-1α protein stability is regulated by the PHD-mediated proline hydroxylation in HIF-1α and consequent VHL-dependent proteasomal degradation [30,31]. To examine whether minoxidil affected the HIF-regulating pathway, parental VHL-deficient renal carcinoma cell line UMRC2 was treated with minoxidil and the experiment was repeated in UMRC2/VHL, which was stably transfected with Flag-VHL [32]. The levels of HIF-1α protein were compared in both cell lines. As shown in Figure 3C, minoxidil increased the HIF-1α level only in UMRC2/VHL. No further induction of HIF-1α was observed in UMRC2, while a greater level of HIF-1α was present due to VHL deficiency. While these data support a claim that the minoxidil induction of HIF-1α is dependent on VHL, it remains to elucidate how minoxidil intervenes in VHL-dependent HIF-1α regulation. Since VHL-dependent HIF-1α degradation is initiated by the PHD hydroxylation of HIF-1α, we examined whether minoxidil interfered with the PHD action. To assess PHD activity, an in vitro VHL capture assay [33,34] was performed using a biotinylated HIF peptide with a conserved proline residue susceptible to PHD-mediated hydroxylation. As shown in Figure 4A (Left panel), minoxidil reduced the binding between the HIF peptide and VHL in a concentration-dependent manner, representing minoxidil inhibition of PHD and subsequent HIF hydroxylation. To rule out the possibility that minoxidil impaired VHL binding to hydroxylated HIF peptide, the same experiment was repeated using a chemically synthesized hydroxylated peptide. As shown in Figure 4A (Right panel), the binding of VHL with hydroxylated HIF peptide was not impaired at all by 5 mM minoxidil. These data strongly suggest that minoxidil directly acts on PHD as an inhibitor. To validate this argument in cells, HaCaT cells were treated with minoxidil in the presence of the proteasome inhibitor MG132, and the hydroxylated status of minoxidil-induced HIF-1α was monitored. As shown in Figure 4B, lower levels of hydroxylated HIF-1α were detected in minoxidil-induced HIF-1α than in MG132-induced HIF-1α, despite greater levels of HIF-1α in minoxidil-treated cells.

### 2.4. Millimolar Minoxidil Inhibition of PHD Is Reversed by Ascorbate, a Cofactor of PHD

**Figure 5:**
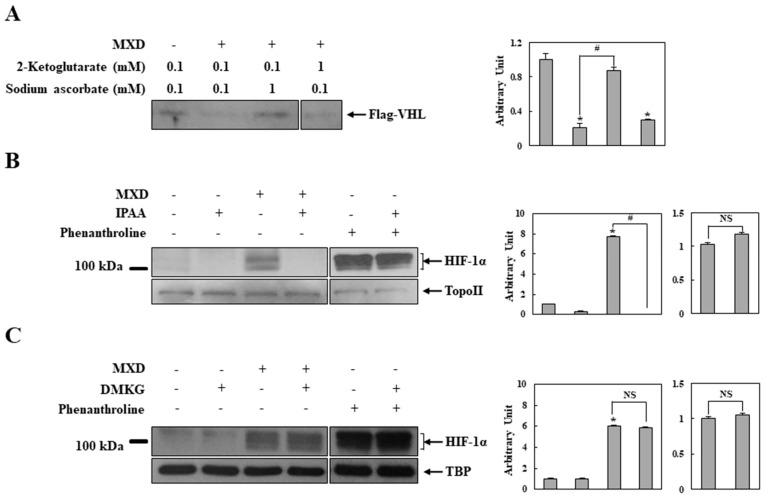
Minoxidil inhibition of HIF prolyl hydroxylase is reversed by ascorbate. (**A**) A VHL capture assay was performed with minoxidil (MXD, 2 mM) in the presence of various concentrations of ascorbate or 2-ketoglutarate, and the resultant blot was probed with an antibody to Flag (VHL) (*n* = 5). * *p* < 0.05 versus untreated group, # *p* < 0.05; (**B**,**C**) HaCaT cells were treated with MXD (2 mM) or phenanthroline (50 μM) in the presence of a cell-permeable ascorbate, (+)-5,6-*O*-isopropylidene-l-ascorbic acid (IPAA, 1 mM); (**B**) or dimethyl 2-ketoglutarate (DMKG, 1 mM); (**C**). HIF-1α in the nuclear extracts was analyzed by Western blot (*n* = 5). * *p* < 0.05 versus untreated group, # *p* < 0.05, NS: not significant. Western blot results are representative of five independent experiments. TBP: TATA binding protein, Topo II: Topoisomerase II.

We investigated how minoxidil inhibited PHD. Since most known PHD inhibitors suppress enzyme activity by interfering with the normal functions of the cofactors [35,36,37], the in vitro assay was performed with minoxidil in the presence of increased concentrations of ascorbate or 2-ketoglutarate. As shown in Figure 5A, VHL association was substantially recovered in the presence of 1 mM ascorbate but not 1 mM 2-ketoglutarate. A 1 mM concentration of ascorbate or 2-ketoglutarate alone did not affect VHL association (Figure S2). To confirm the cofactor effects, HaCaT cells were treated with minoxidil in the presence of a cell-permeable ascorbate or 2-ketoglutarate and nuclear HIF-1α was monitored. To ensure the specificity of the cofactor effects, the experiment was repeated with phenanthroline that inhibits PHD by chelating iron in the active site of the enzyme. As shown in Figure 5B,C, in parallel with the in vitro assay results, the ascorbate abrogated the effect of minoxidil on HIF-1α induction (Figure 5B) while 2-ketoglutarate did not exert an influence on the minoxidil effect (Figure 5C). In addition, the phenanthroline-induced HIF-1α level was not affected by the cofactors (Figure 5B,C).

### 2.5. Millimolar Minoxidil Promotes Angiogenesis in CAM Assay

**Figure 6:**
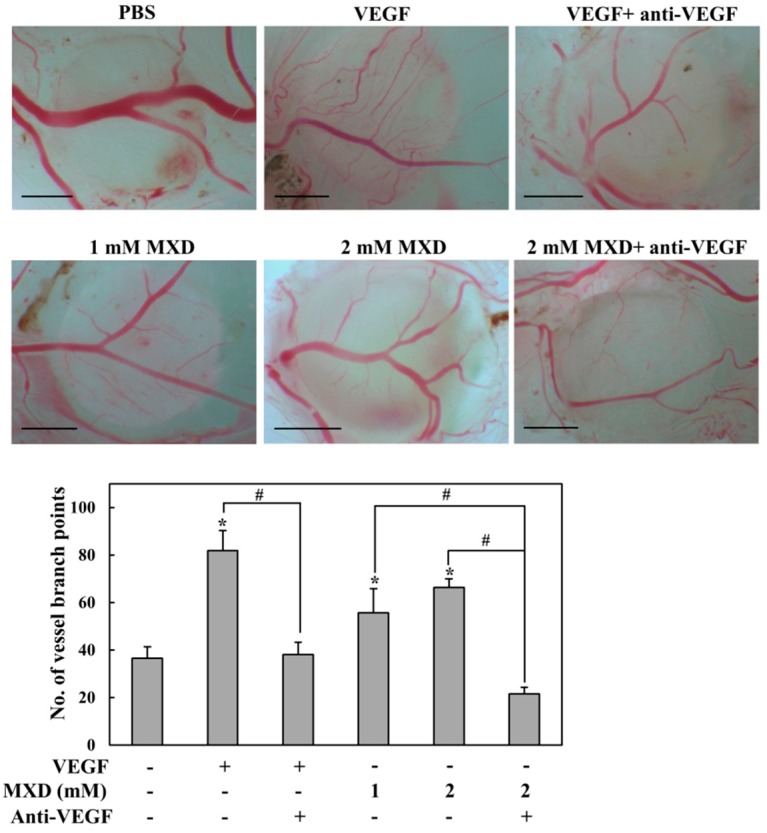
Millimolar minoxidil promotes angiogenesis. A chorioallantoic membrane (CAM) assay was performed in the presence of minoxidil (MXD), VEGF (20 ng/mL), VEGF (20 ng/mL)/VEGF-neutralizing antibody (anti-VEGF, 20 ng/mL), or MXD/anti-VEGF (20 ng/mL) and phosphate buffered saline (PBS) was used as a negative control. The number of vessel branch points was counted by two observers in a double-blind manner. **Upper** panel: Representative images of angiogenesis. The scale bar at the bottom left of each image represents 2 mm. **Lower** panel: Vessel branch points were quantified. The data represent the mean ± SEM (*n* = 5) * *p* < 0.05 versus untreated group, # *p* < 0.05.

Although millimolar minoxidil activated an angiogenic pathway, HIF-1-VEGF, it was unclear whether minoxidil could promote angiogenesis. To examine this, millimolar minoxidil was subjected to the chorioallantoic membrane (CAM) assay, an in vivo angiogenesis test. The same experiment was performed in the presence of a neutral antibody against VEGF (anti-VEGF) to verify VEGF dependency. As shown in Figure 6, although the angiogenic effect of minoxidil was not as great as that of VEGF alone, minoxidil promoted angiogenesis. Moreover, the effect of minoxidil was completely abrogated by a pretreatment with VEGF-neutralizing antibody.

## 3. Discussion

In this study, the molecular effect of millimolar minoxidil, which may be relevant to minoxidil-mediated hair growth, was investigated. In human skin cells, millimolar minoxidil induced VEGF, a possible hair growth factor, in a HIF-1-dependent manner. Minoxidil inhibited PHD by interfering with the normal function of ascorbate, a cofactor of the enzyme, leading to a stabilization of HIF-1α protein and a subsequent activation of HIF-1. In an in vivo angiogenesis assay, millimolar minoxidil increased blood vessel formation in a VEGF-dependent manner.

Since VEGF is an effector for hair growth, molecular mechanisms underlying minoxidil-mediated VEGF induction have been investigated to reveal the minoxidil pharmacology for hair growth [4,5,11]. Most of the studies were performed at low micromolar concentrations of minoxidil, likely based on the fact that hair growth is a side effect of minoxidil administered orally for the treatment of hypertension, which affords a low micromolar concentration of minoxidil in the blood [5]. However, topical application, which is the administration method for the treatment of hair loss, likely achieves millimolar levels of minoxidil in the scalp skin [22,23,25,26,38].

Unlike recent papers demonstrating that low micromolar minoxidil induces VEGF via adenosine receptors [11], our data showed that 8-SPT, a specific adenosine receptor antagonist, did not prevent a millimolar minoxidil induction of VEGF, implying that topically applied minoxidil may utilize (an) additional mechanism(s) for VEGF induction.

We suggest that millimolar minoxidil upregulates the secretion of VEGF via the activation of HIF-1. This argument is supported by the data showing that (1) millimolar minoxidil induced HIF-1α; and (2) the minoxidil induction of VEGF was inhibited by a specific HIF-1 inhibitor, echinomycin, and was significantly attenuated in HIF-1α^−/−^ mouse embryonic fibroblasts. This is not surprising given that *VEGF* is a typical target gene of HIF-1. Moreover, our data showing that topical application of 5% minoxidil increased VEGF and HIF-1α in mouse skin support the pharmacologic notion of the cellular effects.

PHD may be a direct and major molecular target for topical minoxidil-mediated VEGF induction, probably leading to hair growth. We showed that millimolar minoxidil inhibited PHD in an in vitro VHL binding assay. Consistent with a minoxidil inhibition of PHD resulting in the prevention of VHL-dependent HIF-1α degradation [18], minoxidil induced HIF-1α in cells with functional but not with defective ubiquitin E3 ligase VHL, and the disappearance of HIF-1α protein was substantially delayed in cells pretreated with minoxidil. A Western blot analysis showing that minoxidil effectively prevented the hydroxylation of cellular HIF-1α strengthens our argument. Although potassium channel opening is a major pharmacologic effect of minoxidil, diazoxide, another potassium channel opener, did not induce HIF-1α, ruling out the involvement of the channel in HIF-1α induction. In parallel with this result, minoxidil sulfate (100 and 200 μM), the active metabolite of minoxidil that acts on the potassium channel [5,39], did not inhibit PHD or induce HIF-1α in the skin cells. This molecular effect may place minoxidil in a unique position among potassium channel openers that stimulate hair growth, including diazoxide, pinacidil, and minoxidil [5]

Minoxidil inhibition of PHD was reversed by ascorbate, a cofactor of the enzymes, and the ascorbate effect was confirmed in cells. Cell-permeable ascorbate abrogated the minoxidil induction of HIF-1α. Since PHD is inhibited when the iron in the active site is chelated with polyphenolic compounds [35,37], we suggest that minoxidil inhibition of PHD occurs via interrupting ascorbate binding to iron. The transient association of the required factors, ascorbate and 2-ketoglutarate, with the iron in the active site of PHD is crucial for the hydroxylation of its substrate HIF-1α [27,40]. Structurally, minoxidil possesses amines and a nitric oxide moiety for chelation with metals such as iron. The structural feature of positioning amines adjacent to nitric oxide may confer the ability of millimolar minoxidil to chelate iron, thereby inhibiting PHD. However, given that millimolar minoxidil was required for an effective inhibition of PHD, the potential chelating moiety is not potent compared with typical chelating moieties, such as catechols, that have low micromolar IC_50_ [35,37,41].

Given that oral minoxidil, which cannot achieve a millimolar level of minoxidil in the scalp skin, and topical diazoxide (3%) are able to promote hair growth [5,42], it is not clear, for now, how much the cellular effects by millimolar minoxidil contribute to topical minoxidil-mediated hair growth. Further investigation is needed to clarify the pharmacological role of the cellular effects in hair growth.

Collectively, minoxidil topically applied to the scalp may activate an angiogenic pathway HIF-1-VEGF, a potential positive pharmacologic effect for hair growth, by inhibiting PHD.

## 4. Materials and Methods

### 4.1. Chemicals and Animals

Minoxyl solution (5% minoxidil) and its vehicle were gifted from Hyundai Pharm. Co., Ltd. (Seoul, Korea). Minoxidil (6-Piperidin-1-ylpyrimidine-2,4-diamine 3-oxide), 8-(*p*-sulfophenyl)theophylline hydrate (8-SPT), echinomycin, diazoxide, cycloheximide, sodium 2-ketoglutarate, sodium ascorbate, and ferrous chloride were purchased from Sigma Chemical Co. (St. Louis, MO, USA). (+)-5,6-*O*-Isopropylidene-l-ascorbic acid was purchased from Tokyo Kasei Kogyo Co. (Tokyo, Japan). All other chemicals were reagent-grade, commercially available products. Male Institute for Cancer Research (ICR) mice (25~30 g, 7 weeks old) were purchased from Samtako Inc. (Gyeonggi-do, Korea) and were housed in conventional cages, acclimatized for 3–7 days under standard light- and climate-controlled conditions with free access to food and water. The animal protocol used in this study has been reviewed and approved by the Pusan National University–Institutional Animal Care and Use Committee (PNU-IACUC) on ethical procedures and scientific care (Approval No.: PNU-2013-0325, 25 May 2013).

### 4.2. Cell Culture

The methods used for isolating and culturing human dermal papillar cells (DPCs) have been described previously [43]. DPCs were cultured in Dulbecco’s modified Eagle’s medium (DMEM; Hyclone, South Logan, UT, USA) with 10% fetal bovine serum (FBS; Hyclone, South Logan, UT, USA) containing 100 units/mL of penicillin and 2.5 μg/mL of fungizone. Third to fourth-passage DPCs were used. Human keratinocytes (HaCaT cells), human renal cancer UMRC2 cells, UMRC2/VHL cells (stably transfected with VHL) [32], and mouse embryonic fibroblast cells (wild-type and HIF-1α^−^/^−^) (Gift from Dr. Neckers, NIH/NCI, Bethesda, MD, USA) were grown in DMEM (Hyclone) supplemented with 10% FBS and penicillin/streptomycin (Hyclone).

### 4.3. Topical Treatment with Minoxidil

Minoxidil (25 μL of 5% Minoxyl solution/cm^2^) or its vehicle was applied to the dorsal area of ICR mice, which is an experimental method to examine minoxidil effects in skin [44,45]. The animals were randomly grouped (untreated, vehicle-treated, and minoxidil-treated), and one group consisted of five mice. Before application, the hair of the dorsal skin was shaved carefully. The mice were sacrificed by CO_2_ 4 h after the topical application, and all skins were biopsied and homogenized to prepare tissue lysates for the VEGF analysis and tissue nuclear lysates for the Western blot of HIF-1α.

### 4.4. Immunoblot Analysis

Cells were lysed, and nuclear extracts prepared as described in [46]. To prepare tissue nuclear extracts, the mouse skin was removed, mixed with a 6-fold amount of buffer C (10 mM Hepes (pH 7.9), 10 mM KCl, 0.2 mM EDTA, 0.3 μM aprotinin, 1 μM pepstatin, and 1 mM PMSF), and homogenized. NP-40 (10%) was added to the homogenates at a ratio of 50 μL/mL after a 20-min incubation on ice, and the mixture was vortexed for 15 s and centrifuged at 16,800 *g* and 4 °C for 3 min to afford the nuclear pellets. After removing the supernatants, an appropriate volume of buffer N (20 mM Hepes (pH 7.9), 0.4 M NaCl, 1 mM EDTA, 0.3 μM aprotinin, 1 μM pepstatin, and 1 mM PMSF) was added to the nuclear pellets and the tubes were rotated at 4 °C for 20 min followed by centrifugation at 16,800 *g* and 4 °C for 15 min. Protein concentrations in the lysates were determined by the BCA method. Tissue and cellular extracts were electrophoretically separated using 7.5 or 10% SDS-PAGE gels. HIF-1α and hydroxylated HIF-1α protein were detected in nuclear extracts (50 μg for tissue and 20 μg for cell) using a monoclonal anti-HIF-1α antibody (BD Biosciences Pharmingen, San Jose, CA for human HIF-1α or Novus Biologicals, Littleton, CO for mouse HIF-1α) and a monoclonal anti-hydroxylated HIF-1α antibody (Cell Signaling Technology, Danvers, MA, USA), respectively. Experiments were performed and normalized with antibodies to topoisomerase II (Topo II, Santa Cruz Biotechnology, Dallas, TX, USA) or TATA binding protein (TBP, Abcam, Cambridge, MA, USA). Western blot images were quantified by Image Lab software (version 5.2 build 14, Bio-Rad, Hercules, CA, USA).

### 4.5. In Vitro VHL Capture Assay

Biotinylated wild-type or proline-hydroxylated peptides (corresponding to HIF residues 556–574) were synthesized (Peptron. Daejeon, South Korea), dissolved in sterile water (500 μg/mL), and incubated with streptavidin beads (Pierce ImmunoPure, Rockford, IL, USA) at 4 °C for 2 h. An in vitro VHL capture assay was performed using the HIF peptides immobilized on streptavidin beads as described previously [41]. Beads (2 µg peptide/20 µL) suspended in reaction buffer (20 mM Tris pH 7.5, 5 mM KCl, and 1.5 mM MgCl_2_) were aliquoted into separate tubes, and the reaction buffer was added along with cofactors (100 µM 2-ketoglutaric acid, 100 µM l-ascorbic acid, and 50 µM ferrous chloride). Then, any inhibitors or competing factors were added to the appropriate tubes. A 5 µL aliquot of in vitro translated (IVT, Promega, Madison, WI, USA) PHD-2 was added to the reaction mixtures for 1 h at 30 °C. Subsequently, the beads were washed with VHL binding buffer (20 mM Tris pH 8, 100 mM NaCl, 1 mM EDTA, and 0.5% NP40) followed by addition of 10 µL IVT Flag-VHL for 2 h at 4 °C [34]. The beads were washed, SDS Laemmli buffer was added, the samples were boiled, subjected to SDS-PAGE, and the resultant blots were probed for Flag.

### 4.6. CAM Assay

For the CAM assay, the procedure was same as described elsewhere [47]. Briefly, fertilized eggs were procured from Siprigoal Poultry Farm (Gyeongbuk, Korea) and were incubated at 37 °C by maintaining 55% relative humidity. After 10 days, the eggs were inspected for developing blood vessels, and a hypodermic needle was used to make a hole in the wider part of the egg. To create a false air sac, a second hole was made at the broad part of each egg by applying negative pressure from the wider part. A window of 1 cm^2^ was made in the shell of each egg above the area of the false air sac. Sterile filter disks, which were pretreated with 3 mg/mL of cortisone acetate, soaked with VEGF (20 ng/disk), and dissolved in phosphate buffered saline (PBS) with 1% bovine serum albumin (BSA), were placed on the growing CAMs. Then, the test compounds of different concentrations and vehicles were pipetted onto the filter disk placed on the CAM. Eggs were returned to the incubator after sealing the hole and window with Scotch tape. After 72 h of incubation, the CAM tissues underneath the filter disk were resected from the embryos and then harvested. The number of blood vessels sprouting from the main vessel in a circular region covered by the filtered disk was placed under microscope, pictured, counted, and analyzed by using an image software program.

### 4.7. VEGF Analysis

Cells were treated as indicated in the figure legends. Medium was collected following 10 h treatment. VEGF was analyzed using a VEGF ELISA kit (R&D Systems, Minneapolis, MN, USA). An experiment for each condition was carried out in triplicate. To measure tissue VEGF level, the tissue homogenates prepared using buffer C were centrifuged at 400 *g* and 4 °C for 2 min. The supernatants (100 μL) were transferred to fresh microtubes and then centrifuged at 16,800 *g* and 4 °C for 10 min. An appropriate volume of the supernatant was subjected to ELISA. The protein amount in each sample was determined to normalize VEGF levels.

### 4.8. Data Analysis

The results obtained from five separate experiments are expressed as mean ± standard error of the mean (SEM). One-way ANOVA followed by Tukey’s HSD test was used to test the difference between the data, and the post-hoc tests were run only if F achieved *p* < 0.05 and there was no significant variance inhomogeneity. Differences with *p* < 0.05 were considered significant. Sigmastat (SPSS Inc., Chicago, IL, USA) was used for the statistical analysis of the data.

## Supplementary Materials

Supplementary materials can be found at http://www.mdpi.com/1422-0067/19/1/53/s1.
