# Supplementary material for: Minoxidil Induction of VEGF Is Mediated by Inhibition of HIF-Prolyl Hydroxylase
Source: Int J Mol Sci. 2017 Dec 25;19(1):53. doi: 10.3390/ijms19010053 (PMC5796003; doi:10.3390/ijms19010053)
Supplement: Supplementary file 1 [file ijms-19-00053-s001.pdf]

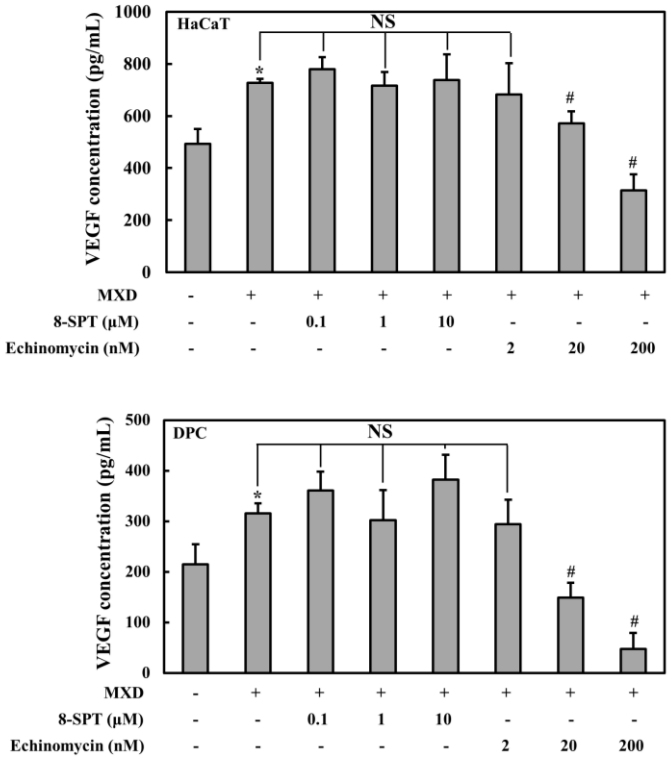

Figure S1. Dose response for 8-SPT and echinomycin.

Human keratinocytes (HaCaT cells) were treated with minoxidil (MXD, 2 mM) for 10 h in the presence of various concentrations of 8-SPT or echinimycin, and VEGF secretion was monitored in cell culture supernatants by ELISA (Upper panel). The same experiment was performed with human dermal papilla cells (DPCs, Lower panel). The data represent mean ± \SEM (n=5) $^{*}P < 0.05$ vs. untreated group, $^{\#}P < 0.05$ vs. MXD alone, NS: not significant.

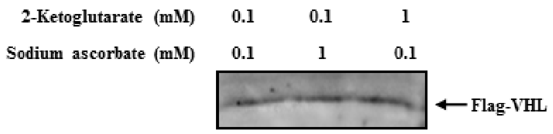

Fig S2. The effect of 1 mM ascorbate or 2-ketoglutarate alone on VHL association.

VHL capture assay was performed in the presence of 1 mM ascorbate or 2-ketoglutarate without minoxidil (MXD) and the resultant blots were probed with the antibody to Flag (VHL).
